# Sea Urchins Predation Facilitates Coral Invasion in a Marine Reserve

**DOI:** 10.1371/journal.pone.0022017

**Published:** 2011-07-18

**Authors:** Rafel Coma, Eduard Serrano, Cristina Linares, Marta Ribes, David Díaz, Enric Ballesteros

**Affiliations:** 1 Centre d'Estudis Avançats de Blanes, Consejo Superior de Investigaciones Científicas, Blanes, Spain; 2 Institut de Ciències del Mar, Consejo Superior de Investigaciones Científicas, Barcelona, Spain; 3 Departament d'Ecologia, Facultat de Biologia, Universitat de Barcelona, Barcelona, Spain; 4 Centre Oceanogràfic de Balears, Instituto Español de Oceanografía, Palma de Mallorca, Spain; National Institute of Water & Atmospheric Research, New Zealand

## Abstract

Macroalgae is the dominant trophic group on Mediterranean infralittoral rocky bottoms, whereas zooxanthellate corals are extremely rare. However, in recent years, the invasive coral *Oculina patagonica* appears to be increasing its abundance through unknown means. Here we examine the pattern of variation of this species at a marine reserve between 2002 and 2010 and contribute to the understanding of the mechanisms that allow its current increase. Because indirect interactions between species can play a relevant role in the establishment of species, a parallel assessment of the sea urchin *Paracentrotus lividus*, the main herbivorous invertebrate in this habitat and thus a key species, was conducted. *O. patagonica* has shown a 3-fold increase in abundance over the last 8 years and has become the most abundant invertebrate in the shallow waters of the marine reserve, matching some dominant erect macroalgae in abundance. High recruitment played an important role in this increasing coral abundance. The results from this study provide compelling evidence that the increase in sea urchin abundance may be one of the main drivers of the observed increase in coral abundance. Sea urchins overgraze macroalgae and create barren patches in the space-limited macroalgal community that subsequently facilitate coral recruitment. This study indicates that trophic interactions contributed to the success of an invasive coral in the Mediterranean because sea urchins grazing activity indirectly facilitated expansion of the coral. Current coral abundance at the marine reserve has ended the monopolization of algae in rocky infralittoral assemblages, an event that could greatly modify both the underwater seascape and the sources of primary production in the ecosystem.

## Introduction

Natural and human-caused disturbances can trigger the fall of a dominant trophic group of organisms and the rise of another [1]. The relevance of this change to the ecosystem varies. But, if the affected group has an important impact on elemental cycles, the change in composition can affect the flows of energy and materials [2], [3]. In the marine realm, the decline of coral reefs and the shift from coral to macroalgae-dominated communities are the clearest examples of the widespread implications and consequences of these changes [4]–[6]. In contrast, the dominance of macroalgae in the rocky shallow infralittoral zone is a common pattern in temperate marine environments [7] where they represent the primary source of energy and organic matter [8]. Macroalgae usually represent the dominant trophic group on Mediterranean infralittoral rocky bottoms [9], although suspension feeders (e.g., mussels, some polychaetes) can occasionally outcompete algae in enriched (eutrophic) waters [10]–[12]. Native zooxanthellate corals (e.g., *Cladocora caespitosa*) can also constitute the dominant trophic group [13], [14]. However, the exotic coral *Oculina patagonica* (De Angelis D'Ossat 1908) has become widespread in the Mediterranean [15]–[17] since its discovery in 1966 in the Gulf of Genova (Italy) [18], which challenges present conceptual framework [9].

Populations of *O. patagonica* were first described in 1973 as isolated colonies at some locations in the western Mediterranean. Abundant populations were observed only in areas highly affected by humans [19]. Later reports have discovered populations in natural habitats [15], [20]–[22]. Therefore, in addition to its geographical spread in the Mediterranean, the species appears to be increasing in abundance in some areas. This population increase may affect the stability of algae as the dominant trophic group in shallow Mediterranean rocky communities and prompts an investigation into what mechanisms are likely to be involved in the increase of *O. patagonica*.

Short- and long-term changes in shallow Mediterranean communities from natural habitats are known to be regulated by bottom-up mechanisms (nutrient availability, irradiance, catastrophic events) as well as top-down controls (mainly herbivory) [8], [23]–[25]. But the Mediterranean is being affected by the main global change threats (i.e., overfishing, habitat degradation, pollution, species introduction and global warming, [26], [27]). Then, anthropogenic impacts (i.e., nutrient uploads, climate change, overfishing and their associated cascading effects) interact with natural mechanisms to ultimately shape the underwater seascape on most Mediterranean shores. In this context, our understanding of the synergistic effects of global change threats on the dynamics of invasion of exotic species is still scarce. To avoid some of the anthropogenic impacts, mainly overfishing, the study was conducted at a Marine Protected Area (MPA), where management plans permit underwater assemblages to attain and maintain their natural population status [28].

The effects of global change threats on the population dynamics of species are unlikely to be additive but mediated by their biotic interactions [29]. Then, occurrence and determination of the effects of key species is especially relevant. Key species are species that are important to ecosystem structure and function by driving ecosystem processes or energy flow [30]. Although invasion of exotic species is a widespread threat to the integrity and functioning of native ecosystems, the role that key species play in invaded communities is still poorly known. Therefore, a major challenge to our understanding of ecosystem functioning is determining whether a few species have a preponderant role in shaping community composition [31]–[33].

The pattern of dominance of macroalgae in shallow habitats from temperate ecosystems is especially evident in the rocky shallow infralittoral zone from oligotrophic seas such as the Mediterranean [23], where erect algae dominate [9]. The only exception to this pattern occurs under extreme physical disturbance and/or high sea urchin densities wherein encrusting coralline algae predominate [34], [35].

In the Mediterranean, the reduction of fish abundance is one of the main factors causing changes in the structure of rocky infralittoral assemblages [36]–[38]. However, the grazing activity of fishes, mostly *Sarpa salpa* do not create open spaces and/or coralline barrens [34]. The most important biological perturbation that generates open space in Mediterranean shallow rocky habitats is herbivory by sea urchins [24], [39]–[41]. Grazing activity by sea urchins can remove algal canopies and/or prevent their recovery, providing and maintaining cleared patches in the substratum on which other organisms can settle and survive [25], [42]. Mediterranean herbivorous fishes play a secondary role in shaping infralittoral assemblages (but see [43], [44]), and some predators (e.g., *Diplodus* spp.) even benefit algae by altering the behavior and abundance of sea urchins [45].

Studies of trophic cascades in which sea urchins play a pivotal role have contributed to an understanding of benthic community structure [24], [37], [38], [46]. Therefore, sea urchins, considered a key species in Mediterranean shallow infralittoral ecosystems because they control the growth of seaweed populations [47], [48], may contribute to an understanding of the cause of coral increase. Sea urchin densities seem to be controlled mainly by the abundance of predators, the presence of refuges and resource availability [25], [49]–[51]. Thus, the hypothesis is that an increase in the abundance of a zooxanthellate coral that spatially competes with macroalgae could be mediated by sea urchins through the creation of barren areas that enhance coral settlement or survival.

Other factors that can affect the structure and dynamics of benthic communities such as predation, competition, facilitation, diseases and environmental conditions [52]–[54] should not be disregarded to contribute to the understanding of the coral pattern of variation. They were examined on the basis of our observations as well as from those of other studies in the area (see Text S1 in supporting information, SI).

In order to understand the dynamics of *Oculina patagonica*, in 2002 we started an assessment of the coral population in the shallow infralittoral environments of Islas Hormigas (Murcia, SE Spain), a well-conserved Marine Protected Area (MPA) excluded of major human impacts where *O. patagonica* was already present. The aims of the study were twofold: (1) to examine abundance and the pattern of variation of the coral *O. patagonica* over time in the MPA Cabo de Palos-Islas Hormigas, and (2) to contribute to the understanding of the main mechanisms that may have allowed the coral's abundance and its variation to occur.

## Results

### Density and coverage of *Oculina patagonica* over time

**Figure 1:**
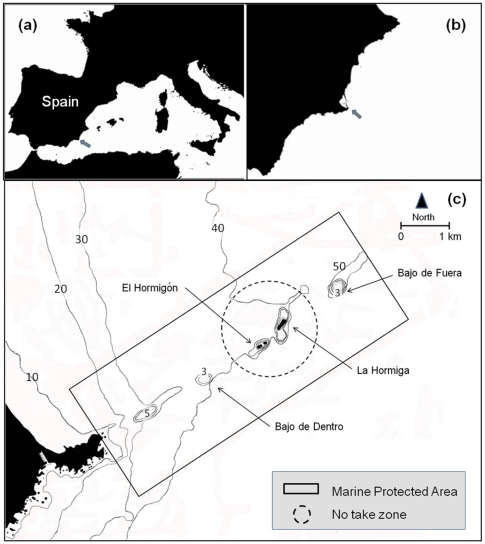
Study sites. (a) Location of Cape of Palos (south-east Spain) in the NW Mediterranean. (b) Location of the Marine Reserve of Cape of Palos-Islas Hormigas. (c) Location of 4 study sites at the Cape of Palos-Islas Hormigas Marine Reserve: Bajo de Dentro, Bajo de Fuera, La Hormiga and El Hormigón.

**Figure 2:**
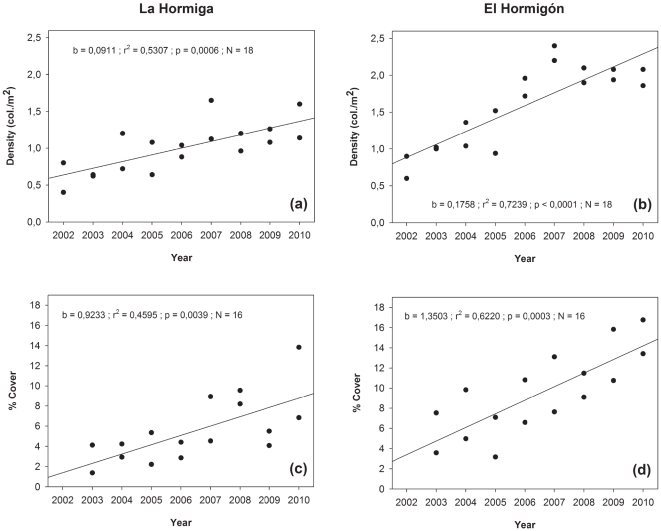
Trends exhibited by the density and the coverage of *Oculina patagonica* over time at La Hormiga and El Hormigón. Pearson product moment correlations between coral density and time and between coral coverage and time are indicated.

The density of coral colonies of *O. patagonica* increased at La Hormiga and El Hormigón (Figure 1) over the study period (2002–2010; Figure 2a,b). Mean density varied from 0.60 to 1.37 colonies m^−2^ at La Hormiga and from 0.75 to 1.97 colonies m^−2^ at El Hormigón. These measurements represent an average density increase of 0.091±0.021 (slope ± SE) and 0.176±0.027 colonies m^−2^ year^−1^ (Figure 2a,b), respectively, resulting in total increases of 128% and 163% for each respective location over the 8 year time period (Figure 2a,b).

The proportion of surface bottom occupied by *O. patagonica* varied from 2.75 to 10.34% at La Hormiga and from 5.55 to 15.09% at El Hormigón. These variations represent an average increase in cover of 0.923±0.267% per year (slope ± SE) and 1.350±0.281% per year (Figure 2c,d), respectively, resulting in total increases of 276% and 172% for each respective location over the 7 year time period (2003–2010, Figure 2c,d).

### Size structure of *O. patagonica* over time

The increase in mean colony size between 2003 and 2010 was not statistically significant [El Hormigón: p = 0.0704, N = 8; La Hormiga: p = 0.1063, N = 8, Table 1]. The coefficient of variation (SD/mean) did not vary over time (El Hormigón: 1.52±0.23, mean ± SD, p = 0.3453, N = 8; La Hormiga: 1.71±0.39, p = 0.9315, N = 8).

**Table 1:** *Oculina patagonica*.

Locality	Year	Area	N	-------------- Colony size (cm^2^) ------------------				kurtosis (g2)			%Ni colonies
		(m^2^)		Mean	SE	Min.	Max.	g2	SEg2	sig(>2)	<100 cm^2^
Hormigón	2003	100	98	417.2	63.9	4.9	4128.3	18.05	0.48	37.36	27.55
	2004	50	60	617.0	99.1	15.9	3848.5	5.23	0.61	8.60	21.67
	2005	100	123	416.7	43.5	19.6	3068.0	8.05	0.43	18.59	17.89
	2006	100	184	473.1	63.8	9.6	7854.0	40.11	0.36	112.53	28.26
	2007	100	230	451.1	50.0	7.1	7854.0	45.23	0.32	141.50	26.96
	2008	80	160	514.4	63.7	12.6	5345.6	15.63	0.38	40.99	18.75
	2009	100	201	661.6	80.2	7.1	8576.8	24.16	0.34	70.78	17.91
	2010	100	197	766.2	79.7	0.8	6013.2	7.24	0.34	21.01	17.26
Hormiga	2003	100	63	436.7	117.6	15.9	7088.2	42.95	0.59	72.20	19.05
	2004	50	48	372.8	63.0	19.6	2164.8	8.50	0.67	12.61	16.67
	2005	100	86	434.5	79.3	19.6	6361.7	50.37	0.51	98.01	25.58
	2006	100	95	382.3	49.0	4.9	2375.8	4.85	0.49	9.90	35.79
	2007	80	111	485.3	101.6	4.9	8251.6	32.81	0.46	72.10	29.73
	2008	100	108	822.3	158.4	7.1	11309.8	19.18	0.46	41.59	16.67
	2009	100	117	409.6	57.1	7.1	5674.5	45.62	0.44	102.81	22.22
	2010	100	137	754.6	109.3	0.8	9940.2	29.94	0.41	72.80	18.25
Bajo Fuera	2002	100	100	392.7	72.8	7.1	6361.7	46.15	0.48	96.49	34.00
	2010	50	148	402.3	44.1	0.8	2827.4	6.34	0.40	16.00	30.41
Bajo Dentro	2002	100	55	262.1	62.9	8.3	2375.8	12.23	0.63	19.30	49.09
	2010	100	231	257.8	24.9	0.2	3318.3	30.91	0.32	96.91	35.50

Descriptive statistics regarding the size distribution of the populations at study sites. Area: sampled area at each site and year; N: number of colonies examined at each site; sig(>): kurtosis is significant if absolute value of coefficient/SE >2.

The proportion of the smallest size class (0–100 cm^2^) over the study period ranged from 17 to 28% at El Hormigón and from 17 to 36% at La Hormiga, indicating the prevalence of small size classes at both locations (Figure S1, Figure S2, Table 1; skewness provided similar information and, therefore, it is not shown). The proportion of the smallest size class exhibited its highest values from 2006 to 2007 at both locations (Table 1). These results indicate that recruitment success of the coral contributed to the density increase observed in both populations during these years. The kurtosis coefficient of the size structure of colonies at both locations showed results that were more peaked than normal distributions (Table 1) which indicates that the change in demographic parameters was recent.

### Sea urchins population over time

**Figure 3:**
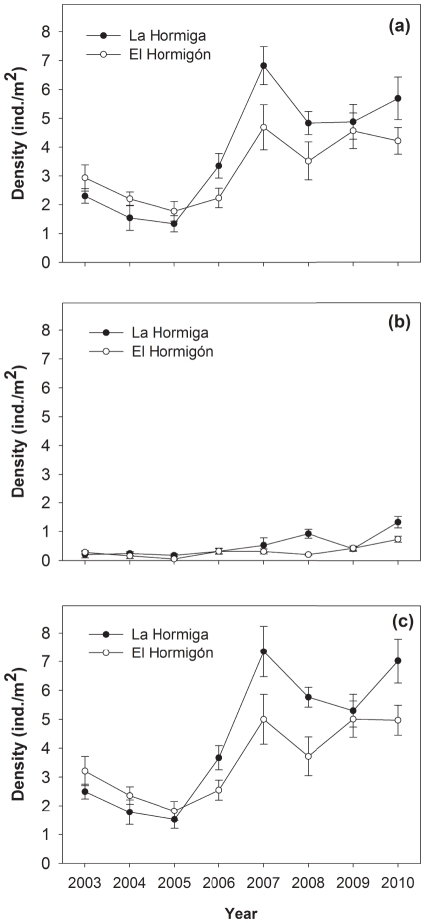
Density of sea urchins (ind m^−2^; mean ± SE) over time. Only sea urchins with >2 cm in test diameter were counted. a) *Paracentrotus lividus*. b) *Arbacia lixula*. c) both sea urchins species together.

Density of urchins increased over time (Time effect, Figure 3, Table 2). However, the pattern of variation over time differed between both species (Time-Species interaction, Table 2). The density of both species was constant and low from 2003 to 2005 (*P. lividus* mean density: 1.73 and 2.05 individuals per m^2^ (ind m^−2^) at La Hormiga and El Hormigón, respectively; *A. lixula* density: 0.14 and 0.21 ind m^−2^ at La Hormiga and El Hormigón, respectively). Density of *P. lividus* increased and then remained constant and high from 2007 to 2010 (mean density: 4.36 and 5.51 ind m^−2^ at El Hormigón and La Hormiga, respectively). This density increase was mainly caused by the high recruitment observed in 2006 and 2007 (Figure 3a). In contrast, the density of *A. lixula* increased steadily from 2006 to 2010 (Figure 3b).

**Table 2:** Summary of a three-way ANOVA comparing sea urchins density among locations (La Hormiga, El Hormigón), time (2003 to 2010) and species (*Paracentrotus lividus*, *Arbacia lixula*).

Effect	df	MS	F	p
Location	1	0.3080	13.17	0.1644
Time	7	0.7637	14.52	0.0011
Species	1	23.9064	20937.67	0.0044
Location × Time	7	0.0526	1.73	0.2427
Location × Species	1	0.0011	0.04	0.8517
Time × Species	7	0.1609	5.30	0.0214
Location × Time × Species	7	0.0304	0.68	0.6856
Error	32	0.0445		
Cochran's test			ns	
Transform			Nil	

The species and time factors were considered as fixed in the analyses and location was randomized.

The abundance of *P. lividus* was about 8 times greater than the abundance of *A. lixula* (mean density 3.52 ind m^−2^ versus 0.41 ind m^−2^, respectively, Species effect, Table 2). Therefore, the pattern of variation in abundance of both sea urchins over time was mainly driven by *P. lividus*. Density varied from 1.46 to 7.02 ind m^−2^ at La Hormiga and from 1.62 to 4.96 ind m^−2^ at El Hormigón, which represent an increase of 381 and 206%, respectively over the 7 years time period, although mainly due to the increase during the 2006–2007 time-period (Figure 3c).

**Figure 4:**
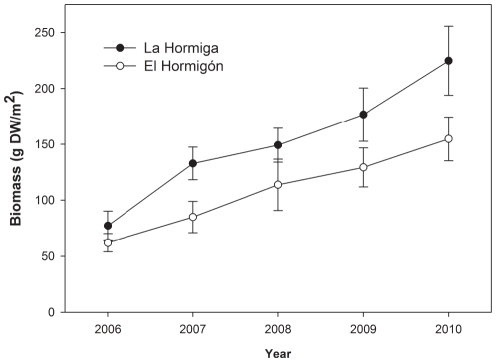
Biomass (g dry weight m^−2^; mean ± SE) of the sea urchin *Paracentrotus lividus* at La Hormiga and El Hormigón between 2006 and 2010.

We studied size structure of *P. lividus* between 2006 and 2010. The highest frequencies of small sea urchins (size class 2, >2–3 cm MTD) were found in 2006 and 2007, suggesting a high level of recruitment in the preceding years (Figure S3). This recruitment appears to form the basis of the overall urchin density increase observed during this time period. However, although density stopped increasing after 2007 (Figure 3c), the biomass of *P. lividus* demonstrated a similar increase over time at La Hormiga and El Hormigón (Figure 4, two-way ANOVA comparing *P. lividus* biomass among locations and time, time effect F_4,10_ = 18.9034, p = 0.0073), mainly due to the increase in mean size of the individuals (Figure S3). This effect was similar in both locations (location-time interaction F_4, 10_ = 0.4040, p = 0.8018).

### Sea urchins and coral abundance

**Figure 5:**
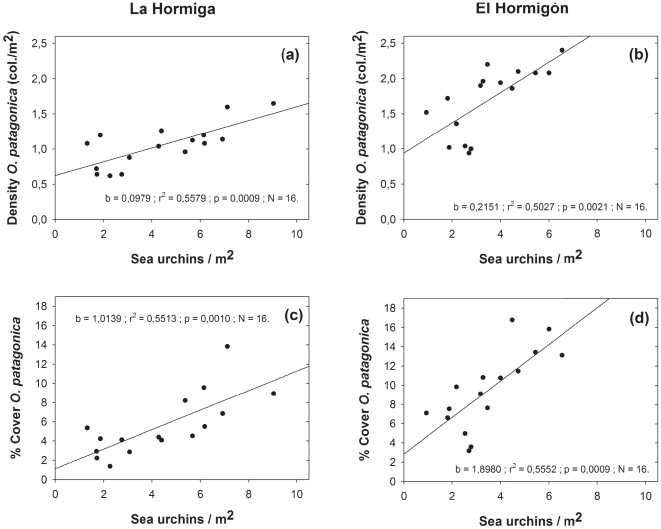
Pearson product moment correlation between the density of both sea urchin species (*P. lividus* and *A. lixula*) and abundance of the coral *Oculina patagonica* at both studied locations . [La Hormiga a) density and c) cover; El Hormigón. b) density and d) cover].

The abundance of *O. patagonica* (density and coverage) at the scale of 50 m^2^ was strongly related to sea urchin densities at La Hormiga and El Hormigón over the study period 2003–2010 (Figure 5).

**Figure 6:**
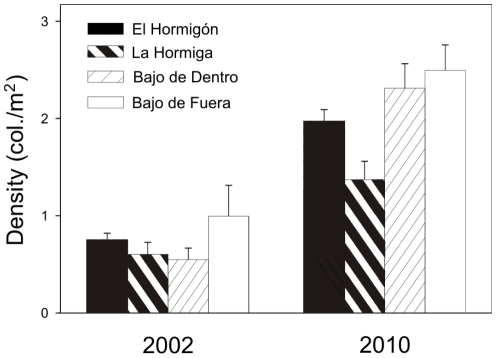
Density of *Oculina patagonica* colonies in 2002 and 2010 at the four studied sites.

In 2002 and 2010, an examination of coral density at two other locations (Bajo de Dentro and Bajo de Fuera, Figure 1) allowed us to determine whether the increase in abundance observed at La Hormiga and El Hormigón was also present at other locations. Density of coral colonies increased over time at all four locations (2-way ANOVA comparing coral colonies density among locations and time, F_1,-_ = 48.057, p = 0.0056, Figure 6). However, the increase in coral colony density did not differ among locations (F_3,3_ = 1.6838, p = 0.3396, Figure 6).

Levels of sea urchin density at Bajo de Dentro (8.6±0.8 ind m^−2^, mean ± SE) and Bajo de Fuera (9.6±0.7 ind m^−2^) were similar to those observed at La Hormiga (7.0±0.8 ind m^−2^), and higher than those observed at El Hormigón (5.0±0.5 ind m^−2^) (one-way ANOVA comparing sea urchins density among locations in 2010, F_3,36_ = 4.9260, p = 0,0057; Scheffe's contrast test).

These results reveal a local-scale pattern of increase in the abundance of both coral colonies and sea urchins. The pattern has occurred in four places that are nearby to each other (within 4 km distance) but separated by 50–80 m deep channel (two of the locations are small islands, La Hormiga and El Hormigón, and the other two, Bajo de Dentro and Bajo de Fuera, are rocky bommies).

### Colony size and presence in open spaces

Open spaces on the substrata were common at La Hormiga and El Hormigón and were covered by encrusting corallines or bare rock. The number of open spaces associated with *O. patagonica* did not differ between the two locations (La Hormiga and El Hormigón; two-way ANOVA comparing abundance of open spaces associated to coral colonies among locations, main effect location: F_1,3,1501_ = 0.0152, p = 0.9093) or over time, despite showing an increasing trend (2005, 2006, 2007, 2010; main effect time: F_3,-_ = 1.4271, p = 0.3886). On average, the mean number of open spaces associated with coral colonies over the entire study period was 3.68±0.23 (SE) per 10 m^2^. The mean size of these open spaces was 0.81±0.34 (SE) m^2^ in 2010. The proportion of space occupied by open spaces (16.0%±1.9; mean ± SE) did not differ between both locations (One-way ANOVA comparing proportion surface bottom occupied by open spaces among both locations, F_1,38_ = 2.4673, p = 0.1245).

**Figure 7:**
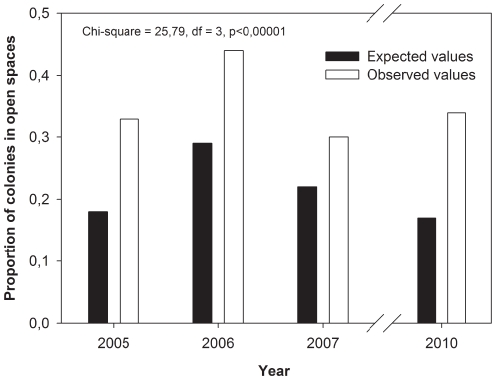
Contrast between the observed proportion of small colonies (up to 100 cm^2^) on open spaces and that expected from the consideration of the abundance of the different colony size classes and their random distribution on open spaces in 2005. 2006. 2007 and 2010.

The contrast between the expected proportion of small colonies (up to 100 cm^2^) associated with open spaces and the observed proportion (see methods) is shown in Figure 7. The observed number of small colonies associated with open spaces was larger than that expected on the four sampled occasions (2005, 2006, 2007 and 2010, Chi-square, X^2^ = 25.79, df = 3, p<0.00001). Thus, small colonies were found to be present on open spaces about 68% more frequently than expected according to random distribution.

## Discussion

### Causes of variation in coral abundance

The increasing abundance of coral colonies of *Oculina patagonica* at the studied MPA from 2002 to 2010 is likely driven by environmental conditions that favor coral's growth. Two main requirements must be met for *O. patagonica* to be able to increase its abundance in a space-limited habitat such as the one in this study: 1) an increase in space availability driven by physical disturbances (i.e., storms) and/or biological interactions (i.e., overgrazing); and 2) the capacity of the species to recruit, grow and survive.

Physical perturbations, such as large storms, can create open spaces [55] such as those observed at the study sites. However, over the study period, open spaces have regularly been observed at the study sites despite a lack of large storms over the study period (authors' observations) [56].

Abundance of the main herbivorous fish species (*Sarpa salpa*) did not vary over the study period [56], nor can they create open spaces [39]. In fact, the most important biological perturbation that generates open space in Mediterranean shallow rocky habitats is herbivory by sea urchins [24], [34]. A threshold of 7–9 adult sea urchins m^−2^ may cause an ecological shift from macroalgae assemblages to coralline barrens [35], [39]. Current sea urchin abundance in the study area (5–9 ind m^−2^) is similar to densities known to cause barrens, and is therefore great enough to be considered a feasible explanation for the open spaces regularly present at our study sites.

The second requirement necessary for coral colonies to increase in abundance is the capacity of the coral species to recruit, grow and survive. Statistical results concerning the size structure of the coral colonies identifies high recruitment as a main factor causing this increase in coral abundance. This result is consistent with evidence that recruitment can also be a critical cause of changes in coral-macroalgae abundance [57]–[59]. However, in this study, recruitment did not result in a decrease in mean colony size (Table 1), which indicates that the species is indeed meeting its requirements for growth and survival.

The polychaete *Hermodyce carunculata* appears to be the main predator of *O. patagonica* in the Mediterranean [60]. The presence and the effects of this worm were observed on very rare occasions during the study (see Text S1). Therefore, predation does not seem to be an important factor affecting the coral populations at our study sites.

Sea surface temperature in the NW Mediterranean is exhibiting a pattern of increase [61] and current evidence indicates that the coral species may benefit from the lengthening of the growing season due to the warming pattern [62], [63]. However, analysis of the SST data showed that lengthening of the growing season did not vary over the study period nor did mean annual temperature (see Text S1). These results are most probably related to the short-term oscillatory pattern that sea water temperature exhibits in the NW Mediterranean [61]. Therefore, the observed pattern in coral abundance can not be attributed to a variation in sea water temperature.

**Figure 8:**
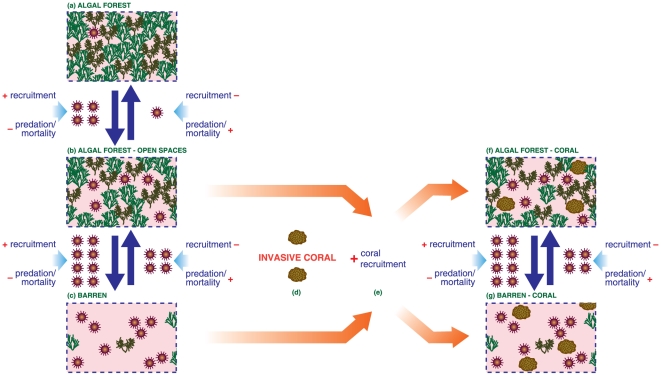
Schematic representation of the observed interactions. The two major assemblages in Mediterranean rocky infralittoral ecosystems are represented at the left side: erect algal forest (a) and coralline barrens (c). Variations in sea urchins density and their grazing impact is the main driver of the shift from algal forests to coralline barrens and *vice versa*. Intermediate densities of sea urchins create and maintain open spaces in the space-limited algal forest (b). These open spaces are usually filled up again by erect algae in a dynamic process of creation and removal of open spaces. However, under the presence of the invasive coral *Oculina patagonica* (d), these open spaces facilitate coral recuitment (e) and increase the abundance of the coral to the extent of matching that of some dominant erect macroalgal species. Therefore, under the presence of *Oculina patagonica* and high to medium sea urchin grazing, two new assemblages flourish: an algal forest-coral assemblage (f) and a coral-coralline barren assemblage (g), depending on the abundance and grazing impact of sea urchins.

At the study area, an increase in sea urchin population density would increase the availability and persistence of cleared patches, the first crucial step for the establishment of coral colonies. This observation is in agreement with the observed relationship between sea urchins abundance and that of *O. patagonica* (Figure 6). Furthermore, the presence of small coral colonies that have settled preferentially on areas cleared by sea urchins (Figure 7) and the size of the cleared spaces provide compelling evidence about the positive relationship between sea urchin density and coral abundance. This result, together with the observed pattern of coral recruitment, implies that the increase in sea urchins abundance is one of the main causes of the increase in density and coverage of coral colonies (Figure 8).

Although a causal relationship cannot be inferred from the statistical correlation observed between the abundance of coral and sea urchins, the existence of the correlation is a proof of concept of the basic idea underlying the hypothesis. It is apparent that sea urchin grazing promotes the recruitment of *O. patagonica* colonies, in accordance with results obtained in coral reef ecosystems [64]–[66]. Thus, interspecific facilitation appears to be one of the main mechanisms involved in the observed increase in abundance of coral colonies (Figure 8). These results highlight the crucial role that herbivory by sea urchins appears to play in increasing the abundance of coral colonies.

The main fish species identified as successful sea urchin predators are the Sparidae *Diplodus sargus*, *Diplodus vulgaris* and *Sparus aurata*, and the Labridae *Coris julis*, *Labrus merula*, *L. viridis*, *Symphodus roissali* and *S. tinca* [67]–[70]. Populations from all these fish species have not varied significantly over the study period [56]. Nutrient levels and the presence of sea urchins refuges did also not vary over the study period [56]. Therefore, recruitment appears to be the primary factor contributing to the increase in sea urchins abundance. Although the factors responsible for large fluctuations in sea urchin abundance remain poorly understood, there is evidence that high level of recruitment can outweigh fish predation [24], [71]. Our study provides evidence that a change in the demography of a sea urchin species can drive a relevant change in community structure. Under unchanged fish predation, nutrients and refuge conditions, the increase of *P. lividus* biomass resulted from both a high recruitment and a good period of growth for sea urchins. Two non-exclusive causes may have contributed to the success of *P. lividus*: i) favourable climatic conditions, and ii) low predation on reproductive populations and on planktonic larvae. However, this study can not distinguish between both causes and, most probably, it may have been a combination of them.

### Relevance of the current coral abundance

The percent cover observed for *O. patagonica* at our study sites (10–15%) was only slightly lower than those reported for total coral cover in coral reef ecosystems (e.g., Great Barrier Reef: 27%, Indo-Pacific: 22%, Caribbean: 7%, Florida Keys: 16%, [5], [6], [72]), emphasizing the importance of this species within the benthic community of this temperate ecosystem.

Macroalgae species composition exhibits regional, bathymetric and seasonal changes in the biomass of the dominant species [23]. Interannual changes have also been documented in relation to species substitution, sea urchin activity and overfishing [24], [73], [74]. However, none of these spatial and temporal variations imply a change in the dominant trophic group (i.e., all changes involve algal species). Even in the case of successfully introduced species, changes in dominant species generally involve the replacement of the dominant algal species by an exotic algae species [75].

Algal assemblages at the study sites were dominated by different species of macroalgae as it is the case in other well-conserved areas in the central western Mediterranean [76], [77]. No relevant changes on relative abundance of the main dominant macroalgae species was observed over the study period but a decrease in abundance of *H. scoparia* (see Text S1).

Detailed data using photo-quadrats [77] in similar shallow infralittoral habitats illustrate that erect macroalgae account for roughly 69.9–91% of surface cover, calcareous encrusting macroalgae account for 28.6–7.5% cover and invertebrates (mainly sponges) account for the remaining 1.5%. Therefore, the current coverage of *O. patagonica* at the study sites is unusual for Mediterranean shallow water assemblages, matching the abundance of several species of dominant erect macroalgae. Thus, *O. patagonica* is able to initiate an important change in community structure and end the monopolization of algae in shallow assemblages, an event that could greatly modify both the underwater seascape and the sources of primary production in the ecosystem.

Despite the differences between the temperate Mediterranean and coral reef environments, the observed processes may be similar to those observed in the Caribbean, where the recovery of *Diadema antillarum* populations is known to have enhanced coral recruitment [65], [66]. However, in Caribbean coral reef communities, as in those in other areas, the positive effects of urchins on coral may be diminished or even negated by increases in coral diseases, temperature-related mortality, and coastal habitat degradation [4], [78], [79]. Like the Caribbean, the Mediterranean is also affected by coastal habitat degradation, rising temperatures and diseases [61], [63], [80], [81]. However, in the western Mediterranean these disturbances appear to be affecting *O. patagonica* less than other suspension-feeders thriving in similar habitats, such as *Cladocora caespitosa* and different species of sponges, which have been severely affected by recent mass mortality events [82]–[84].

Our study describes the processes causing the increase of *O. patagonica* inside a single MPA. However, the increasing number of areas that this coral has been reported in the western Mediterranean [17] suggests that the processes described here could also be underway in other areas. In addition, this growth and expansion could be linked to an increase in sea urchin populations related to changes in the food web directly or indirectly enhanced by overfishing or pollution [24], [46], [48], [74], [85].

Shallow infralittoral rocky bottoms in the Mediterranean are undergoing profound changes that result in the disappearance of important habitat engineering species [25], [74]. These changes are often linked to overfishing [24], habitat destruction [74], invasive species [75], mass mortality events [84], [86], [87] or pollution [88]. In this work, we document that the selective predation by sea urchins on the dominant species (macroalgae) created open spaces that enhanced coral settlement and survival. Therefore, within the conditions of the study, trophic interactions contributed to the success of an invasive coral in the Mediterranean because sea urchins grazing activity indirectly facilitated expansion of the coral (Figure 8). We have also presented evidence that the invasive zooxanthellate coral is growing in abundance to levels completely unexpected in the Mediterranean, an event that challenges the current conceptual framework [9], offering an excellent opportunity to study the mechanisms that sustain present benthic communities in this habitat. Furthermore, we discovered new evidence regarding the crucial role of sea urchins in Mediterranean infralittoral communities by demonstrating that sea urchin grazing activity not only causes changes in algal composition, but also facilitates the expansion of an invasive coral.

## Materials and Methods

### Study area

The study was conducted at the Cabo de Palos-Islas Hormigas Marine Reserve which is located in the southeastern part of the Iberian Peninsula (Cape of Palos: 37°38′01″N, 0°41′04″W).

### Sampling

The density and size of coral colonies of *Oculina patagonica* was assessed at 4 locations (Figure 1) in 2002 and 2010. Yearly assessments of the coral populations were conducted in spring at two locations (La Hormiga and El Hormigón) within the marine sanctuary of the Marine Reserve (where no activities other than scientific research can be conducted since 1995) from 2003 to 2010. Although the species is abundant at depths from the surface to 9 m, the greatest abundance was observed around 6 m [89]. At this depth, two randomly located transects (50 m×1 m) were performed by SCUBA divers. Only colonies with at least 50% of their surface area lying within the belt-transect were counted to avoid boundary effect biases to the spatial sampling method [90].

Within the study area, the colonies of *O. patagonica* displayed a predominantly encrusting growth form with a circular-ellipsoidal shape. The surface area of the colonies was estimated by means of *in situ* measuring of the longest dimension of the colony (length, L) and its perpendicular axis (width, W) with a ruler to the nearest millimeter. The surface area was calculated (S, cm^2^) using the formula S = π[L+W]/4]^2^ according to [15].

The abundance of sea urchins (*Paracentrotus lividus* and *Arbacia lixula*) along the same 50 m^2^ transects was also recorded every year from 2003 to 2010. Sea urchin abundance was recorded in plots measuring 10 m^2^. Between 2006 and 2010 size-structure of sea urchins was also estimated by measuring maximum test diameter without spines (MTD). All individuals larger than 2 cm in test diameter were counted and measured with calipers along the whole transect.

To determine whether coral recruitment was facilitated by the presence of open spaces we examined small coral colonies (up to 100 cm^2^) associated with open spaces (a discrete area deprived of, but bordered by, erect macroalgae). A colony was considered to be associated with an open space if a minimum of 50% of the perimeter of the coral colony was in contact with the open space. We examined whether or not each coral colony within the random transects was associated to an open space on a minimum of a 100 m^2^ in 2005, 2006, 2007 and 2010. The observed number of small colonies associated with open spaces was contrasted to that expected. Expected values were estimated by multiplying the total of colonies associated with open spaces by the proportion that the small colonies size class represents from the overall coral population. Observed and expected values from the four different year assessments was tested using Chi-square.

The size of the open spaces within the transects in contact with *O. patagonica* was estimated in 2010. Percent cover of open spaces was assessed within randomly located 1 m^2^ squares (n = 20) by estimating abundance of open spaces in 20 randomly distributed square meters at La Hormiga and El Hormigón. Each square meter estimate was conducted by adding the estimates of 4 adjacent 0.50×0.50 m quadrats. Quadrats were subdivided into 25 squares (each representing 4% of the quadrat), and the open spaces in each subdivision were recorded.

### Statistical analysis

Variation of coral density over time at La Hormiga and El Hormigón was examined using a Pearson product moment correlation. Variation of coral cover (proportion of surface occupied by coral colonies in each 50 m^2^ transect) over time was examined with the same method. A two-way ANOVA was conducted comparing coral density among 4 locations (La Hormiga, El Hormigón, Bajo de Fuera and Bajo de Dentro) and time (2002 and 2010) to examine whether the abundance of the species varied over the study period at the four locations. Prior to analysis, normality was checked using a Kolmogorov test. Homogeneity of variance was tested using Cochran's test, and whenever necessary, data were transformed [91]. Statistics were performed using STATISTICA 6 software package.

Coral size distribution was analyzed by estimating mean colony size, the coefficient of variation (i.e., standard deviation as percentage of the mean), skewness and kurtosis. Variation of the mean colony size over time (2003 to 2010) was examined using a Pearson product moment correlation. Variation of the coefficient of variation over time was examined with the same method. Skewness and kurtosis coefficients were considered significant if g_1_ per SES (standard error of skewness) or g_2_ per SEK (standard error of kurtosis) was greater than 2 [92].

A two-way ANOVA was used to determine whether the number of open spaces varied between locations (La Hormiga and El Hormigón) and over time. Time was considered to be fixed in the analyses, and location was randomized. A one-way ANOVA was used to determine whether the amount of space occupied by open spaces varied between both locations.

A three-way ANOVA was used to compare sea urchin densities among species (*Paracentrotus lividus* and *Arbacia lixula*), locations (La Hormiga and El Hormigón) and time (2003–2010). The factors of species and time were considered to be fixed in the analyses, and location was random. A one-way ANOVA was used to examine variation in the density of both sea urchin species among the four locations in 2010. Pearson product moment correlation was used to examine the relationship between the abundance of both sea urchin species and the abundance (density and coverage) of *O. patagonica*.

The following equation was used to transform *P. lividus* density and size structure into *P. lividus* biomass:
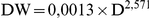
where DW is dry weight in grams and D is the test diameter without spines [35]. A two-way ANOVA was conducted to compare *P. lividus* biomass among locations (La Hormiga and El Hormigón) and time (2006–2010) to examine whether the species exhibited a similar pattern over the study period at both locations. Time was considered to be fixed and location was randomized in the analyses.

## Supporting Information

Figure S1 Size-frequency distribution of *Oculina patagonica* populations between 2003 and 2010 at La Hormiga.
(EPS)

Figure S2 Size-frequency distribution of *Oculina patagonica* populations between 2003 and 2010 at El Hormigón.
(EPS)

Figure S3 Size-frequency distribution of the populations of the sea urchin *Paracentrotus lividus* between 2006 and 2010 from La Hormiga and El Hormigón.
(EPS)

Text S1 Assessment of other factors that may affect the dynamics of the coral and sea urchin populations.
(DOC)
