# Supplementary material for: Sea Urchins Predation Facilitates Coral Invasion in a Marine Reserve
Source: PLoS One. 2011 Jul 18;6(7):e22017. doi: 10.1371/journal.pone.0022017 (PMC3138760; doi:10.1371/journal.pone.0022017)
Supplement: Text S1 — Assessment of other factors that may affect the dynamics of the coral and sea urchin populations. (DOC) [file pone.0022017.s004.doc]

**Supporting information**

**Sea urchins predation facilitates coral invasion in a marine reserve**

**Rafel Coma^1*^, Eduard Serrano^1,2^, Cristina Linares^3^, Marta Ribes^2^, David Díaz^4^, Enric Ballesteros^1^**

**^1^ Centre d’Estudis Avançats de Blanes, Consejo Superior de Investigaciones Científicas, Blanes, Spain**

**^2^ Institut de Ciències del Mar, Consejo Superior de Investigaciones Científicas, Barcelona, Spain**

**^3^ Departament d’Ecologia, Facultat de Biologia, Universitat de Barcelona, Barcelona, Spain**

**^4^ Centre Oceanogràfic de Balears, Instituto Español de Oceanografía, Palma de Mallorca, Spain**

*** Corresponding author. Telephone: 972 336101, Fax: 972 337806, Email: coma@ceab.csic.es**

**Text S1.** **Assessment of other factors that may affect the dynamics of the coral and sea urchin populations**

Here we provide the detailed information on the assessment of other factors that may have affected the dynamics of the coral and sea urchin populations. A summary of each factor assessment is provided in the main body of the study.

Other factors such as physical disturbance, abundance of herbivorous fishes, abundance of sea urchin predators, predation, changes in the main components of the benthic community, and sea water temperature that could have affected the dynamics of the coral and sea urchins populations were examined on parallel studies [1-3] and over the numerous dives conducted at the study area.

**Physical disturbance**

Whether physical disturbance (large storms) caused open spaces in the substrata were recorded on our dives and on the data from the annual monitoring of the MPA. Open spaces have regularly been observed at the study site despite the lack of large storms over the study period (authors' observations) [3].

**Abundance of fish species**

Fish population studies have documented an important increase in abundance of piscivorous fish species at the marine reserve between the establishment of the protection measures in 1995 and 2006 [2]. However, over the study period, the monitoring of fish populations at the study zone have shown that abundance of the main herbivorous fish species (*Sarpa salpa*) and, that of the main fish species identified as successful sea urchin predators (the Sparidae *Diplodus sargus, Diplodus vulgaris* and *Sparus aurata*, and the Labridae *Coris julis, Labrus merula, L. viridis, Symphodus roissali* and *S. tinca* [4-7]) did not vary [3].

**Predation**

The polychaete worm *Hermodice carunculata* is the main described predator of the coral *O. patagonica*. The presence and effects (a clear dotted pattern, see [8]) of the polychaeta on the coral was recorded on our dives. The presence and the effect of this worm were observed on very rare occasions during the study.

**Changes in dominant benthic species**

We estimated the relative abundance of the dominant benthic species of the community using a semiquantitative method at La Hormiga and El Hormigón at about 6 m depth in 2010. This method involves identifying the main species of the community in specific replicated quadrats, and assigning to each species an abundance code based on its contribution to the total coverage. The used codes were: 0: species not present; 1: species <5 % of total; 2: species<10 % of total; 3: species=10-35 % of total; 4: species=35-70 % of total; 5: species>70 % of total. Relative abundances were contrasted with those previously obtained in 2004 during the characterization of this benthic community at La Hormiga and El Hormigón using the same method [1] to determine whether any outstanding change in dominant species composition occurred over the study period.

Characterization of the benthic community of the MPA conducted in 2004 [1] showed that assemblages at the study sites were dominated by different species of macroalgae (*Dictyopteris polypodioides, Sargassum vulgare,* *Neogoniolithon brassica-florida*, *Padina pavonica* and *Halopteris scoparia*). Assessment of the benthic community in 2010 showed the same dominant macroalgae species except for a decrease in abundance of *Halopteris scoparia*.

**Sea surface temperature**

Sea surface temperature (SST) in the NW Mediterranean is exhibiting a pattern of increase [9]. Therefore, sea warming should be considered as a factor that may affect the dynamics of the coral because current evidence indicate that the species may benefit from a lengthening of the growing season of the coral due to temperature increase [10,11].

Daily mean sea surface temperature (SST) from the MPA from January 2003 to December 2010 was examined using satellite measurements by the MODIS (aqua) sensor system, made available as "Ocean Level-2" HDF data by the NASA Goddard Space Flight Center. HDF files were read and processed using Mathlab R2009a software. In the analysis, we only considered high-quality sensor readings of temperature (flag values of 0 or 1), discarding less-reliable readings (flag values of 2 or 3). Suitable SSTs readings (N=1536) used in our analyses corresponded to daily means in a 9 km^2^ area centered at the following coordinates: 37º39'7.09''N--0º39'15.72''W (El Hormigón). Achievement of sea surface temperature of 18ºC has been determined to be a good indicator of summer conditions [11] that favor *O. patagonica* growth. Then, we calculated the number of days that temperature was >18ºC to examine whether lengthening of the summer period has occurred over the time period of the study and may have affected the coral species. We estimated the date in spring that SST was ≥18ºC and the date in fall that it dropped below it on the basis of daily mean values. To avoid bias introduced by short term temperature oscillations, the criteria used to determine these dates was that after the date in spring that SST reached ≥18ºC, daily mean SST values had to remain ≥18ºC at least 80% of the days during the following two weeks. Accordingly, after the date in fall that SST dropped <18ºC, daily mean SST values had to remain <18ºC at least 80% of the days during the following two weeks. Mean annual temperature was also determined.

The number of days that SST was >18 ºC (used as an indicator of summer conditions that favor growth of the species) was 198 ± 13 days/year and did not vary over the study period (r^2^=0.0190, p=0.7446, N=8). Mean annual temperature was 19.31± 0.24 ºC and did also not show a significant change over the study period (r^2^=0.1537, p=0.3366, N=8).

**References**

1. Calvin JC (2004) Caracterización y valoración del hábitat arrecife en el litoral mediterráneo de la región de Murcia. In: JM Gutiérrez-Ortega (ed.) Estudio sobre el estado de Conservación de los hábitats marinos de interés comunitario y/o mediterráneo presentes en el litoral sumergido de la región de Murcia. Consejeria de Agricultura y Agua (Murcia), volumen VI.

2. García-Charton JA, Pérez-Ruzafa A, Marcos C, Claudet J, Badalamenti F, et al. (2008a) Effectiveness of European Atlanto-Mediterranean MPAs: Do they accomplish the expected effects on populations, communities and ecosystems? Journal of Nature Conservation 16: 193-221

3. García-Charton JA, Esparza Alaminos O, Segovia Viadero M, Treviño Oton J, Herrero Pérez A, et al. (2008b) Estudio de seguimiento de la Reserva Marina de Cabo de Palos-Islas Hormigas. Consejeria de Agricultura y Agua (Murcia) y Universidad de Murcia

4. Quignard JP (1966) Recherches sur les Labridae des côtes européennes. Systématique et biologie. Causse et Castelnau, Montpellier.

5. Khoury C (1987) Ichtyofaune des herbiers de posidonies du Parc National de Port-Cros: composition, éthologie alimentaire et rôle dans le reseau trophique. PhD Thesis , Université d'Aix-Marseille II, Marseille

6. Sala E (1996) The role of fishes in the organization of a Mediterranean sublittoral community. PhD Thesis, Université d'Aix-Marseille II, Marseille.

7. Sala E (1997) Fish predators and scavengers of the sea urchin *Paracentrotus lividus* in protected areas of the northwestern Mediterranean Sea. Marine Biology 129: 531-539.

8. Fine M, Oren U, Loya Y (2002) Bleaching effect on regeneration and resource translocation in the coral *Oculina patagonica*. Marine Ecology Progress Series 234:119-125

9. Vargas-Yañez M, Moya MC, García-Martínez ET, Zunino P, Plaza F, et al. (2010) Climate change in the Western Mediterranean sea 1900-2008. Journal of Marine Systems 82: 171-176

10. Rodolfo-Metalpa R, Reynaud S, Allemand S, Ferrier-Pagès C (2008) Temporal and depth responses of two temperate corals, Cladocora caespitosa and Oculina patagonica, from the North Mediterranean Sea. Marine Ecology Progress Series 369: 103-114.

11. Coma R, Ribes M, Serrano E, Jiménez E, Salat J, et al. (2009) Global warming-enhanced stratification and mass mortality events in the Mediterranean. Proccedings of the National Academy of Sciences USA 106: 6176-6181.
